# Middle East respiratory syndrome coronavirus: review of the current situation in the world

**DOI:** 10.1186/s40696-016-0019-2

**Published:** 2016-05-04

**Authors:** Michael Shapiro, Beny London, Daniel Nigri, Alon Shoss, Eyal Zilber, Itay Fogel

**Affiliations:** 1 IDF Medical Corps Training School, City of Training Bases, Israel; 2 IDF Medical Corps, Surgeon General Headquarters, Tel Hashomer, Israel; 3 Department of Pediatrics C, Schneider Children’s Medical Center of Israel, Petach Tikva, 49202 Israel

**Keywords:** MERS-CoV, Coronavirus, Unusual biological event, Outbreaks, Emerging diseases

## Abstract

This article reviews the current epidemiology and clinical presentation of Middle East Respiratory Syndrome Coronavirus (MERS-CoV) infection and describes the preparedness plan of several countries. The MERS-CoV was first reported in 2012 and has since infected more than 1600 patients in 26 countries, mostly in Saudi Arabia and the Middle East. The epidemiology of the infection is compatible with multiple introductions of the virus into humans from an animal reservoir, probably dromedary camels. The clinical presentation ranges from no symptoms to severe pneumonitis and respiratory failure. Most confirmed cases so far were part of MERS-CoV clusters in hospital settings, affecting mainly middle-aged men and patients with a chronic disease or immuno-suppressed status. There is no vaccine or anti-viral medication available. Viral epidemics can occur anywhere in today’s “global village”. MERS-CoV is a relatively new virus, and this work is intended to add to the still-sparse data on its epidemiology, modes of transmission, natural history, and clinical features as well as to describe the preparedness plan for MERS-CoV infection in several countries. Effective national and international preparedness plans are essential to predict and control outbreaks, improve patient management, and ensure global health security.

## Background

Middle East respiratory syndrome is caused by a novel coronavirus (MERS-CoV) first isolated in the Kingdom of Saudi Arabia in 2012 from the respiratory tract secretions of a Saudi businessman who died from viral pneumonia [1]. Health officials first reported the disease in September 2012, when most cases originated in Saudi Arabia and, to a lesser extent, the United Arab Emirates (UAE). Subsequently, cases were identified in patients living outside the Arabian Peninsula and the Middle East, who were infected either during a stay in the Middle East or by close contact with an individual from an endemic country [2]. Although the virus has no gender predisposition, most affected patients have been previously healthy men with a median age of 50 years [3].

## Epidemiology

The origin of MERS-CoV has been widely discussed. Originally, a bat reservoir was posited based on the phylogenetic similarity of certain bat coronaviruses with MERS-CoV [4, 5]. However, the exact MERS-CoV strain found in humans has not been identified in any bat species [6, 7]. Furthermore, in almost none of the known cases to date was there a clear bat source of infection or a consistent history of contact with bats.

Another possibility is the dromedary camel. Dromedary camels have been found to harbor the same MERS-CoV and MERS-CoV-like antibodies as humans and to shed the virus in high numbers in secretions from the upper respiratory tract [8]. Studies of archived secretion samples obtained from camels in Dubai in 2005 revealed neutralizing antibodies to MERS-CoV, indicating that the virus is not new to the Arabian Peninsula. These findings also suggest that MERS-CoV is widespread and has previously infected various camel populations, including some in countries where human cases have yet to be reported [9]. Camels are currently recognized as the main animal reservoir, with primary cases occurring in areas where they are an important part of life. Unpasteurized camel milk may be a route of transmission, but so far there are no data that MERS-CoV is excreted into camel milk [10].

The outbreak of the novel virus among humans was first reported in April 2012, when eight healthcare workers from one intensive care unit in Jordan had respiratory illness; however, the source was unknown at that time [11]. In June 2012, a 60-year-old man presented in Saudi Arabia with acute pneumonia and subsequent renal failure, which eventually led to his death. A previously unknown coronavirus was detected in his sputum and provisionally called human coronavirus Erasmus Medical Center (hCoV-EMC) [1]. Shortly thereafter, in September 2012, the same virus was detected in a patient with severe respiratory illness who had been transferred from the Middle East to the United Kingdom. Combining with retrospective analysis of stored samples from the aforementioned outbreak in Jordan, the novel virus, renamed MERS-CoV, was recognized as the cause of a new severe respiratory disease [12].

The epidemiology of MERS-CoV infection since isolation of the pathogen in 2012 is compatible with multiple introductions of the virus into humans from the animal reservoir, with no long-term sustained human-to-human transmission [13]. The recent epidemic might be explained by progressive changes that have taken place in camel farming in Saudi Arabia, leading to a large increase in the population of camels and in camel farms in the proximity of cities [14].

On March 23, 2016, the World Health Organization (WHO) published a report on 1698 laboratory-confirmed cases of MERS-CoV infection [15]. The case-fatality rate was approximately 36 % [15]. All cases were directly or indirectly linked through residence or travel to 10 countries: Saudi Arabia, the UAE, Qatar, Jordan, Oman, Kuwait, Yemen, Lebanon, Egypt, and Iran [15]. There are also reports of sporadic export of the disease to countries in four continents, including the United Kingdom, France, Tunisia, Italy, Malaysia, Greece, Austria, Turkey, the United States of America, Germany, Philippines, and Thailand [15–17]. The largest outbreak of the virus outside its endemic region began in May 2015, with the first-ever confirmed case in South Korea, in a patient who had recently visited several countries in the Arabian Peninsula. Over the next 2 months, 186 additional cases were confirmed, including the first in China, with a total of 36 deaths. The last confirmed case in this region was recorded in July 2015 [18].

## Clinical presentation and risk factors

The median incubation period of MERS-CoV is 5 days (range 2–14 days) during which time the host remains asymptomatic. The clinical presentation of the disease is wide, ranging from no symptoms to mild upper respiratory symptoms such as fever, cough, and myalgia to severe pneumonitis and respiratory failure. Gastrointestinal symptoms are also common, including loss of appetite, abdominal pain, nausea, vomiting, and diarrhea. Less common manifestations are hemoptysis or diarrhea without fever [19, 20].

Some studies suggest that diabetes, hypertension, chronic heart disease, and chronic kidney disease are risk factors for MERC-CoV infection and a severe clinical presentation [19]; however, this theory needs to be further investigated. The majority of confirmed cases were MERS-CoV cluster cases in hospital settings, and the main risk factors were male gender, underlying chronic disease, immunosuppressive status, and hospital stay [19]. The male predominance may reflect Middle East societal practices, which heighten the likelihood of exposure in males relative to females, or a higher rate of certain underlying medical conditions among older men.

There has been a recent increase in reports of asymptomatic or mild MERS-CoV infection among women and children. However, there are no corresponding findings on contact tracing among healthcare workers or close contacts of infected patients in German, UK and US hospitals, or in serologic surveys of blood derived from donors and abattoir workers in endemic regions in 2012 [21]. Therefore, it is hard to estimate the proportion of asymptomatic patients among infected individuals.

Currently, no vaccine or anti-viral medication is approved for the treatment of patients with MERS-CoV infection, and primary treatment is supportive [22]. Nevertheless, new vaccines are under research and development and some candidates are paving their way for phase-I trials in humans [23–25]. These include a vaccine based on the major surface spike (S) protein using recombinant nanoparticle technology, a full-length infectious cDNA clone of the MERS-CoV genome in a bacterial artificial chromosome, a recombinant modified vaccinia Ankara vaccine expressing full-length MERS-CoV spike protein, and vaccines encoding the full-length MERS-CoV S protein and the S1 extracellular domain of S protein using adenovirus vectors.

## National preparedness plans and public health response for MERS-CoV infection

The emergence of new unknown virus in conjunction with high fatality rate of the disease has led to major public health and international concern [15]. The WHO guidelines apply a relatively sensitive definition of suspected cases and emphasize the importance of a high index of clinical suspicion for diagnosis owing to the high mortality rate. Some countries, especially those in endemic regions, have developed their own preparedness and response plans which are based on WHO and Centers for Disease Control and Prevention (CDC)’s recommendations. The Oman’s Ministry of Health implemented a national plan which was based on strengthening five pillars of action, including: (1) public health surveillance and contact management. Field visits were conducted to every confirmed case by the national public health services and exposed individuals were monitored for 14 days after the last exposure; (2) building laboratory capacity, including diagnostic capacity with primers for MERS-CoV testing, and training laboratory personnel on triple-packing and shipment of samples. Furthermore, training to first responders and intensivists on how to collect nasopharyngeal samples; (3) infection prevention and control, including mask-fit testing for all healthcare workers who could be involved in patient care; (4) case management; and (5) risk communication [26]. The government of the Republic of Korea summoned a Rapid Response Team following the outbreak in their country. The team was composed of infectious disease specialists and infection control professionals, and they established national guidelines for the diagnosis and management of MERS-CoV infection. Together with the epidemiology investigation team of the local government, control strategies were discussed, which included: (1) contact tracing; (2) surveillance of polymerase chain reaction testing of healthcare workers and patients according to their level of contact; (3) preemptive isolation of pneumonia cases; (4) environmental disinfection; and (5) cleaning and enforcing the use of personal protective equipment (PPE) among healthcare providers [27]. The possibility of MERS-CoV occurring in Israel is high given Israel’s geographic location in the Middle East and the thousands of Israeli Moslems who make the pilgrimage to Mecca (the Hajj) each year. Therefore, the Israel Ministry of Health (IMOH) has drafted preparedness guidelines that generally follow the CDC guidelines, although the CDC did not include Israel among the countries at risk. These guidelines recommend laboratory evaluation for all healthcare workers with a severe acute respiratory illness of unknown etiology, and in cases of clusters of severe respiratory symptoms of known etiology. In addition, the approval of the public health services is required before any case may be designated a suspected MERS-CoV infection, thereby ensuring early involvement on a national level in every instance of the disease. Information regarding MERS-CoV was disseminated by the distribution of leaflets and placement of informative posters at Ben-Gurion International Airport and three land-border crossings between Israel and Jordan [28].

## Case definition

The probable case definition of MERS-CoV infection is based on clinical and epidemiological criteria, and it is dynamic, subject to modification as more data become available. According to the current, updated definition, MERS-CoV is suspected in a patient with fever and acute respiratory symptoms who meets the following epidemiological conditions: contact with a patient with a confirmed diagnosis, either directly or through clinical samples, within 14 days prior to symptom onset, or a visit to a country in or near the Arabian Peninsula within 14 days prior to symptom onset [15]. The diagnosis is confirmed by laboratory confirmation.

## Laboratory diagnosis

The CDC recommends the collection of multiple specimens from different sites, including upper respiratory tract, lower respiratory tract and serum, at different times after symptom onset [29]. Laboratory diagnosis is based on the detection of the virus in upper respiratory and lower respiratory samples by using real-time reverse transcriptase polymerase chain reaction (RT-PCR) assay. This detection is performed by targeting upstream regions of the E gene (upE) or within open reading frame (ORF)1b, using upE for screening and ORF1b for confirmation. Another real-time RT-PCR assay targeting the MERS-CoV nucleocapsid protein gene has been developed and can be used for screening and confirmation. When there are discordant results between two real-time RT-PCR assays, the sequencing of an amplicon generated from an appropriate RT-PCR assay should be performed to confirm test results. Lower respiratory tract specimens have been found to be more sensitive than upper respiratory tract specimens for the detection of MERS-CoV. In addition, the development of a rapid diagnostic kit is important for the timely diagnosis of suspected MERS-CoV cases. Serological testing can be used to screen the contacts of infected patients and to retrospectively confirm MERS-CoV infections [30]. Virus isolation in cell culture is not recommended at this time since it requires laboratory working under high biosafety level [29].

## Infection control

Some epidemiological data support the possibility of both contact and airborne transmission of coronaviruses; however, the modes of transmission of MERS-CoV specifically are not completely understood. There is no consensus regarding precautionary recommendations for MERS-CoV; whereas the WHO recommends the contact and droplet precautions for all suspected cases and airborne precautions only for aerosol-generating procedures [31], the CDC advocates the use of airborne and contact precautions for all patient care activities [32]. Several countries have taken the hard line of the CDC guidelines and recommend that direct healthcare workers should place all patients with a suspected infection in an isolated negative-pressure room and take adequate precautions by wearing two pairs of gloves, a disposable gown, and a face shield, and donning an N-95 respirator [28, 33, 34]. Currently, all suspected and confirmed cases should be treated in hospitals regardless of their medical condition, however transportation of patients to hospital requires additional emphasis, taking into account the small space of the vehicle and the close contact with the patient. The few guiding principles are that patients should be transported on a dedicated mission with the minimum number of crew members and should wear a surgical mask, if tolerated. The direct healthcare providers should wear the aforementioned PPE against contact and airborne transmission and avoid cough- or aerosol-generating procedures unless necessary. In addition, all transportations of patients should be coordinated with public health services [35].

## Recommendations for travelers to and from affected countries

The WHO guidelines do not include a travel warning for countries endemic for MERS-CoV. However, it urges travelers to refrain from contact with persons with acute respiratory symptoms, maintain vigilant personal hygiene, avoid eating uncooked food or unpasteurized milk, especially from camels, and avoid contact with animals and their excretions. Travelers from endemic countries in whom fever and acute respiratory symptoms develop within 14 days after their arrival should contact their physician as soon as possible [15].

## Discussion

The recent MERS-CoV epidemic and the outbreak of severe acute respiratory syndrome coronavirus (SARS-CoV) in 2003 emphasized that new emerging diseases can occur at any time and in any place without prior notice. MERS-CoV shares certain similarities with SARS-CoV: both are zoonotic viruses which belong to the genus *Betacoronavirus* and can cause an acute respiratory illness which may be fatal. Although MERS-CoV has a higher case-fatality rate than SARS-CoV (36 vs. 10 %), both viruses are highly pathogenic and can apparently be transmitted through all routes of exposure. Moreover, currently, there are no effective vaccines or antiviral treatments for these diseases [36]. Failure to contain the outbreak in its first stages can have devastating effects, emphasizing the importance of a national preparedness plan for unusual biological events (UBEs). There are some key elements to improve the readiness for UBEs such as MERS-CoV and SARS-CoV. (1) *Containment abilities* Implementation of quarantine measures to isolate healthy people who have been in contact with infected people and designated hospitals for receiving sick patients. (2) *Infection control* Given the unknown route of transmission, PPE against contact, droplet and airborne transmissions should be allocated to first responders. (3) *Epidemic management team* The need for a multidisciplinary team to advise the Ministry of Health regarding modes of action has been shown to be essential in the last outbreaks. Nevertheless, this consulting team should have routine training sessions in order to be ready during outbreaks. (4) *Epidemiologic investigation team* An early epidemiologic investigation is vital for containment of an outbreak and it allows early intervention that could prevent morbidity and mortality. Skilled teams with advanced epidemiologic investigation tools can detect earlier the index case and take prompt countermeasures. (5) *Table-top and “real-life” exercises* Exercises should be undertaken for testing contingency plans and coordination between organizations. The Israeli Orange Flame preparedness build-up project, in which medical and nonmedical staff are trained at three levels: tactical, operational and strategic, is a good example for a comprehensive drill that is aimed at improving national readiness and preparedness for emerging diseases [37].

## Conclusions

Viral epidemics can occur anywhere in today’s “global village”. MERS-CoV is a relatively new virus. Effective national and international preparedness plans are essential to predict and control outbreaks, improve patient management, and ensure global health security.

### Take home massages

1. MERS-CoV infection is a zoonotic disease characterized clinically by fever and severe respiratory symptoms. It is most likely transmitted through contact with camels, which are in widespread use in the Arabian Peninsula, where most cases originate, or through contact with sick patients.
2. Many countries have issued preparedness and detection guidelines for healthcare workers and for travelers to endemic countries.
3. No cure for MERS-CoV is currently available. Early detection, appropriate infection-control measures, and supportive treatment are key elements in preventing the next outbreak.

## Abbreviations

MERS-CoV: Middle East Respiratory Syndrome Corona Virus
UAE: United Arab Emirates
WHO: World Health Organization
CDC: Centers for Disease Control and Prevention
PPE: personal protective equipment
IMOH: Israel Ministry of Health
RT-PCR: reverse transcriptase polymerase chain rection
upE: upstream region of the E gene
ORF: open reading frame
SARS-CoV: severe acute respiratory syndrome coronavirus
UBE: unusual biological event
